# Human Responses to Magnetic and Hypomagnetic Fields: Available Evidence and Potential Risks for Deep Space Travel

**DOI:** 10.3390/life15111766

**Published:** 2025-11-18

**Authors:** Rustem R. Kaspranski, Vladimir N. Binhi, Ivan V. Koshel

**Affiliations:** Federal Medical-Biological Agency, Federal Scientific and Clinical Center for Space Medicine and Biology, 24 Suschevskiy Val, Moscow 127018, Russia; kaspranski@mail.ru (R.R.K.);

**Keywords:** biological effects of magnetic fields, human organism, geomagnetic field, hypomagnetic field, interplanetary magnetic field, space, magnetobiology

## Abstract

The growing body of biomedical research reveals that many biological processes are governed by quantum physical principles, including the effects of weak magnetic fields (MFs) at or below geomagnetic strength. Given that life evolved within the geomagnetic field, its significant decrease—the hypomagnetic field (hypoMF)—may disrupt fundamental biological processes. This is particularly relevant for interplanetary missions, where astronauts will encounter prolonged hypoMF conditions alongside other spaceflight stressors. This mini-review synthesizes current knowledge on hypoMF effects, comparing terrestrial and extraterrestrial MF conditions and evaluating evidence from human studies. The initial database search identified 645 records. After most were excluded for various reasons, only 44 publications on the effects of MFs on the entire human body were included in the review. An effect of the hypoMF was reported in 10 of these studies and was absent in 4. Despite some methodological limitations in the available research, the evidence suggests that the human body is not indifferent to hypoMF exposure. We also discuss leading mechanistic molecular hypotheses—particularly the radical pair mechanism. Finally, we identify urgent research priorities to elucidate hypoMF’s biological role and develop countermeasures for future deep space exploration. Addressing these gaps is essential for safeguarding astronaut health and advancing magnetobiology as a frontier discipline in biophysics.

## 1. Introduction

The biological effects of weak magnetic fields (MFs) are now a well-established fact and have been described in numerous monographs [1,2,3]. It has been shown that MFs can trigger various phenomena in organisms, including toxic effects [4,5]. In 2011, the International Agency for Research on Cancer conducted an analysis of several epidemiological studies and concluded that low-frequency background MFs exceeding 0.35 μT are a possible carcinogen to humans [6]. Natural disturbances of the geomagnetic field caused by solar flares are of the same order of magnitude and induce diverse biological responses [7,8,9,10,11,12,13,14]. It appears that prolonged human exposure to altered or fluctuating MFs, compared to the geomagnetic field, is not indifferent and statistically exerts adverse effects on humans.

Magnetic biological effects exhibit high variability due to their dependence on a wide range of physicochemical and physiological conditions. Because of this, they have low experimental reproducibility, which complicates their study. There is currently no evidence that the effects of strong and weak MFs consistently differ in magnitude. Remarkably, both very strong and very weak MFs—including their absence—induce biological effects of the same order of magnitude in organisms (e.g., similar-scale changes in oxidative stress markers or gene expression levels).

It is well known that the Earth acts as a magnet, i.e., it possesses its own MF with a magnitude of about 50 μT. This field is relatively weak, so we usually do not notice it in everyday life. For comparison, the field generated by a standard medical MRI scanner exceeds the geoMF by tens of thousands of times. Conversely, additional MFs experienced by users of household electronics, as well as passengers in automobiles and electric vehicles, measure tens to hundreds of times weaker than the geoMF [15].

Biological tissues exhibit diamagnetic properties overall. A response to MFs, arising from the diamagnetism of constituent atoms and molecules, is extremely weak. The relative magnitude of these physical effects is on the order of $10^{-6}$ [16]. For this reason, it was long believed that organisms were insensitive to MFs.

Although isolated attempts to use MFs in therapy date back over a century [17], it was only relatively recently, approximately 50–60 years ago, that robust evidence established living cells can respond to electromagnetic fields and MFs through behavioral changes—that is, in ways characteristic of living systems [18,19,20,21]. These responses disappear when cells cease functioning as living systems.

Thus, it appears that MFs act not on tissue molecules per se, but rather on biological processes, even though the primary targets are magnetic moments of microparticles—particularly those of unpaired electrons. This phenomenon originates at the atomic–molecular level and is fundamentally quantum in nature (see e.g., [22]).

**Figure 1:**
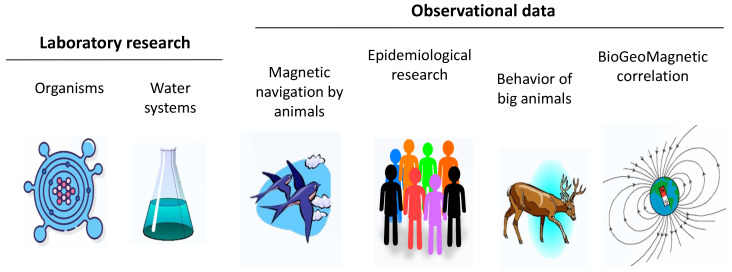
Sources of evidence for weak MF effects on organisms—from single cells to large animals and humans.

It has been established that various organisms, including humans, can respond to minute changes in MFs, as small as approximately one-thousandth of the geoMF. These phenomena include (see Figure 1) animal magnetoreception [23,24,25] and cardiovascular responses to magnetic storms [13,26]. Such effects have also been observed in laboratory animals [12,27,28]—though infrequently, as nonspecific effects generally exhibit random behavior already at the molecular level. Their impact is often masked by various uncontrolled physicochemical factors [29]. However, these phenomena become statistically significant in large-scale epidemiological studies investigating the correlation between background MFs and disease incidence [30].

Effects triggered by substantial weakening of the geoMF—by factors of 100 or more—are of particular interest [31]. Such fields are termed *hypomagnetic* or hypoMFs. Laboratory suppression of the geoMF induces changes in biochemical parameters and behavior across diverse organisms: from bacteria and fungi to mammals and humans; see, e.g., [32,33,34,35]. Human studies remain scarce due to the technical complexity of generating controlled hypoMF environments, but existing data suggest detrimental trends in neurophysiological function. Beyond the biochemical effects of mT-level static MFs [36], hypoMFs induce biological responses at the level of gene expression [37,38,39,40,41,42] and enzyme activity [43,44,45,46,47]. At a higher level, they also alter cardiovascular regulation and cognitive performance.

A study on abstract magnetic moment dynamics [48] showed that magnetic effects in hypoMFs are several times stronger than in weak alternating MFs. A critical concern is the biological consequences of chronic hypoMF exposure and the potential synergy between hypoMF, cosmic radiation, and microgravity, which could exacerbate health risks in space.

The aim of this review is to provide a foundational understanding of (i) the occurrence of hypoMFs in space, (ii) their effects on the human organism, and (iii) potential molecular mechanisms of hypoMF effects—with particular emphasis on their implications for deep space missions. Indeed, if artificial hypoMFs elicit diverse biological responses on Earth, why should not naturally occurring hypoMFs in deep space be considered a potential adverse factor in spaceflight [49,50,51,52,53]?

The structure of the review is consistent with the aforementioned objectives. Section 2 describes the literature selection method. Section 3 provides essential information on the magnitudes of hypoMF in space, on the Moon, and on Mars; as well as the distribution of studies on the biological effects of hypoMF across countries and over time. Section 4 is dedicated to a detailed description of the effects of hypoMF on the human organism. This narrow topic is embedded within the broader discourse on the biological effects of MFs with different spatiotemporal organizations. Unique data are presented on large-scale systems that allow for the exposure of the entire human organism to weak MFs, including hypoMF. Section 5 presents the minimally necessary information on the state of theoretical research into the molecular mechanisms of the biological effects of weak MFs. Section 6 contains a discussion of the peculiarities of hypomagnetic effects and their connection with other harmful factors of a deep space mission. The general conclusions are formulated in Section 7.

## 2. Methods

This work, conducted to map the extant literature, was performed in the spirit of a PRISMA-compliant scoping review [54]. The literature search for this work utilized *Dimensions*, an international database with extensive coverage (100–140 million records), and the Russian database *eLibrary* (20–30 million records). For context, while *Dimensions*’ coverage surpasses that of other major databases like *Web of Science* (60–80 million) and *Scopus* (80–100 million), it requires more rigorous quality control.

A protocol for the review was not registered a priori because the methodology was straightforward and followed standard, transparent steps. The search strategy employed combinations of keywords including ‘human + magnetic + field + effect’, ‘human + hypomagnetic’, ‘human + low + magnetic’, ‘human + weak + magnetic’, alongside their corresponding Russian-language equivalents. The initial database search conducted on 26 August 2025 identified 645 records. A significant proportion of the retrieved records were subsequently excluded for various reasons.

After duplicates and articles that appeared on the list due to random keyword matches were removed, 570 records remained. Additional screening allowed for the exclusion of a further 460 publications. Excluded were articles on magnetic resonance, biomagnetism, the biological effects of moderate and strong MFs, the effects of MFs on animal and plant organisms, engineering solutions, theoretical studies, and review articles. All of these are, evidently, not directly related to the topic of the review—the effects of weak MFs on the entire human body. Publications on the epidemiological effects of weak MFs and correlations between geomagnetic disturbance and characteristics of the human body were also excluded. As is known, these observational studies do not provide information on cause-and-effect relationships. However, minimally necessary references to review publications on these topics, which are required for the correct positioning of the effects of weak MFs on the human body within the structure of scientific knowledge, are provided.

**Figure 2:**
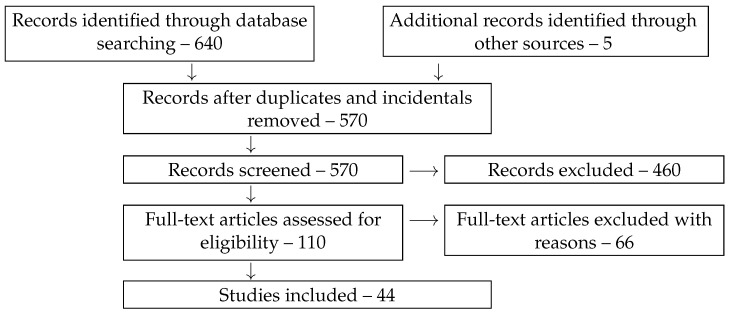
PRISMA flow diagram of the study selection process.

The remaining 110 full-text articles were assessed for eligibility in greater detail. The reasons for further exclusions were as follows: exposure to strong MFs; duplication of work under different titles; duplication in another language; variations of epidemiological studies; unavailability of the source; lack of peer review, i.e., publications in the form of preprints and conference abstracts; further instances of random keyword matching; animal studies; biochemical studies on cells; theoretical and engineering studies; review studies without original data; and methodological inadequacy of works in which authors reported biological effects of picoTesla-level MFs without due instrumental control. As a result, the number of studies included amounted to 44 units, as shown in Figure 2.

We examined these articles, summarized their results, and present them in a concise tabular format supplemented by extended descriptions for particular articles. Data pertaining to whole-body human magnetic exposure systems, as extracted from these studies, are also compiled in a table. The main limitation of this review is its focus on English and Russian sources, which potentially introduces a language bias and excludes significant research disseminated in other languages.

The review incorporates essential background information on the hypoMF, methods for its generation, and the molecular mechanisms of its biological action. Without this information, the review of the current state of knowledge regarding the biological effects of the hypoMF on the human body would lack necessary comprehensiveness.

## 3. Hypomagnetic Field

The MF in the space around Earth is determined mainly by Earth’s intrinsic geoMF and the flux of charged particles from the Sun. As a result of their interaction, a complex magnetic structure is formed—the magnetosphere.

For rough estimates of the MF in near-Earth space, a common approximation treats the geoMF as a dipole field, which decays with distance. For example, at the International Space Station’s orbit altitude (408 km), the MF strength averages about 0.8 of Earth’s surface geoMF, while its direction flips during each orbit revolution. At distances comparable to or exceeding Earth’s radius, the geoMF decreases rapidly—following an inverse-cube law—plummeting below 10 nT beyond 100,000 km. In interplanetary space, the ambient MF (2–80 nT, [55], p. 396) becomes three to four orders of magnitude weaker than the surface geoMF. Such hypoMF conditions are recognized as a potential factor affecting crew health and operational precision during extended lunar/Mars missions [49].

Data on the lunar MF were obtained through analysis of lunar rock samples during the *Apollo* program, as well as from magnetometric measurements of the *Lunar Prospector* and *Kaguya* missions [56]. The crustal MF of the Moon is inhomogeneous, with spatial gradients on the order of 1 nT per degree [57]. Measurements are complicated by magnetic noise from solar radiation. At 30 km altitude above the lunar surface, the magnetic induction is extremely weak, typically about 1 nT, with rare anomalies where the MF reaches 10 nT or higher. Near the surface, proximity to rocks with heterogeneous remanent magnetization can produce localized MFs of several hundred nT.

The Martian MF is below a few microtesla. It exhibits a complex dynamic structure with small- and large-scale inhomogeneities ranging from tens to several thousand kilometers in size. Overall, the Martian MF can be described as a quasi-uniform distribution of local magnetic anomalies, where field induction varies from 1–5 nT to 20–40 nT, with some anomalies reaching up to 1600 nT at 100 km altitude [58].

**Figure 3:**
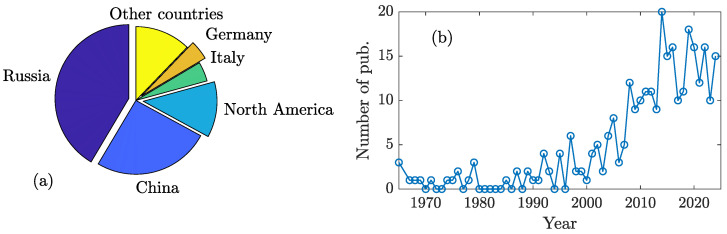
(**a**) Country participation in hypoMF biological effects studies, measured by the number of publication authors (2017–2024). (**b**) Annual worldwide count of English- and Russian-language publications on hypomagnetic research since 1965.

In 2017, a review of 137 experimental studies on the biological effects of hypoMF and their potential molecular mechanisms was published [31]. The studies were selected from approximately 200 available at that time based on methodological quality criteria. During the subsequent period from 2017 to 2024, over 100 additional publications appeared. The country distribution of these publications’ authors is shown in Figure 3a.

Russia, China, and North America serve as the primary “investors” of research in this field, accounting for approximately 80% of published studies. Notably, Russian and Chinese publications significantly outnumber North American contributions. This disparity likely stems from the absence of conclusively established molecular mechanisms underlying these phenomena, and consequently, the predominantly fundamental nature of current research in this domain [59]. These factors limit immediate practical applications, while Western countries prioritize investments in applied research and development.

However, recent years have seen a marked acceleration in this research field (Figure 3b). State-of-the-art laboratories are now identifying specific genes involved in biological responses to hypoMFs [38,39,40,41,42,60,61,62,63]. Current evidence demonstrates that combined exposure to radiation and MF can produce synergistic effects exceeding the sum of individual exposures [64,65,66,67], though not universally [68]. Since the same molecular mechanisms underlie biological responses to both hypoMF and other weak MF types, similar synergistic effects should occur during combined exposure to ionizing radiation and hypoMF. This hypothesis has received direct experimental confirmation [51,69,70,71].

Theoretical progress has been more modest, yet ongoing research into the biophysical mechanisms underlying hypoMF bioefficacy provides grounds for optimism regarding imminent breakthroughs [22]. Successful resolution would identify the primary biophysical target of MF action in organisms. Current hypotheses suggest this target may involve cellular biopolymerization processes, particularly ribosomal protein translation [72]. This complex multistage enzymatic mechanism generates spin-correlated radical pairs during translation—known as magnetosensitive intermediates [73].

## 4. The Human Body’s Response to MF and hypoMF

MF effects in biology are studied both in vitro and in vivo, with key distinctions between these approaches. At the molecular level, MFs modulate sensitive reactions, thereby altering biochemical regulators and biomolecular functions. In vitro studies enable precise control of environmental parameters (temperature, pH, viscosity) and exposure conditions (field homogeneity, magnitude, frequency), simplifying mechanistic interpretation. In vivo systems, however, transform primary molecular responses into complex systemic reactions involving the nervous, immune, and endocrine systems, making causal relationships harder to establish.

Several reviews have addressed the effects of MFs on the human body, but they primarily focus on either relatively strong MFs (>1 mT), ELF/RF alternating MFs, pulsed MFs, wideband background fields, or other exposure combinations [74,75,76,77]. These reviews nevertheless overlook the effects of weak quasi-static MFs, including hypoMFs under whole-body exposure conditions—a critical gap given the permanent hypomagnetic environment in space.

Initial attempts to synthesize data on weak MF effects on humans revealed an almost complete lack of scientific evidence by the 1980s, despite numerous fragmented reports [78]. Today, 45 years later, research on this topic remains scarce—likely due to the high costs of such studies and the absence of well-reproducible effects.

In an early study [79], researchers claimed that humans could orient toward their home location after being displaced dozens of kilometers. The involvement of the geoMF was evidenced by preserved homing ability in blindfolded subjects, which was disrupted when a permanent magnet was placed near their head. However, subsequent studies disputed the existence of this human homing capability [80], Ch. 27–32 in [81].

Numerous epidemiological studies provide more robust evidence, generally confirming associations between human health and background electromagnetic field levels [6,82]. However, such studies may contain systematic biases and only establish correlations without elucidating causal inference. In contrast, laboratory studies on human exposure to MFs—particularly hypoMFs—remain relatively scarce. This scarcity stems from the technical challenges of generating controlled, whole-body MF exposures suitable for prolonged human occupancy [83]. Consequently, most research measuring human psychophysiological responses has been limited to localized MF exposure, typically targeting the head [84,85,86,87,88,89,90,91]. A critical methodological limitation arises as different head regions (and body parts) experience MFs of varying magnitudes and orientations, complicating result interpretation.

Notably, the discussed effects of weak MFs during head exposure fundamentally differ from those of strong, inhomogeneous pulsed MFs used in transcranial magnetic stimulation. In the latter case, the biological effect arises from induced electric fields localized to small cortical areas, sufficient to trigger neuronal firing.

Only a limited number of studies have investigated the effects of weak, moderately uniform MFs on the human body. Achieving adequate field uniformity requires the use of large-scale exposure systems [92].

Table 1 summarizes characteristics of selected magnetic exposure systems designed for studying weak MF effects on humans. These systems vary in size, number of axes, compensation type, and other parameters. The table includes only compensation-based systems, as constructing and operating large mu-metal magnetic shielding systems presents prohibitive technological challenges [83], along with methodological difficulties in designing experiments with unambiguous interpretability.

As shown, no exposure system has yet been developed that meets all requirements for reliable hypoMF research on humans—specifically, a system that simultaneously markedly exceeds body dimensions, supports hypomagnetic conditions, and incorporates active three-axis compensation of ambient magnetic noise.

There are not many studies on the effects arising from exposing a large part of the human body to an MF. In [43], reversing the direction of a static MF with a magnitude comparable to the geoMF led to a reduction in human night vision acuity: the number of errors in recognizing a contrast stimulus increased by a factor of 2–3. In [94], the effect of a 9 h exposure to a 50 Hz, 10 μT MF on the pineal gland function of 32 young men was studied. Analysis of blood plasma samples showed that melatonin secretion did not change under these conditions. In contrast, Wood et al. [95] found that exposure to a 50 Hz, 20 μT MF led to a statistically significant half-hour delay in the rise of nocturnal melatonin concentration in 6 out of 30 men. Seven young men participated in an experiment [98] involving 8-h exposure to a 16.7 Hz, 200 μT MF. The melatonin level in saliva remained unchanged.

In [104], it was found that a pulsed 200 μT MF did not affect basic human perception but could increase pain thresholds in 70 subjects. No details were provided about the pulse waveform. Later, an fMRI study involving 30 participants demonstrated that a similar pulsed sequence altered fMRI patterns during the processing of thermal pain stimuli [105]. In [106], muscle tremor was assessed in 24 subjects exposed to a 1 mT pulsed MF under a double-blind, sham-controlled protocol. The authors reported that the effects were “minor” and that subjects with high-amplitude tremor appeared more sensitive to MF exposure. However, interpreting these findings is challenging because the authors did not specify the MF switching rate, thus neglecting potential electromagnetic induction effects. The observed effects might not stem from the MF itself but rather from eddy currents induced in brain tissue. Due to this ambiguity, many studies on the effects of pulsed MFs on humans are not discussed here; a comprehensive review can be found in [90].

In the study by [88], the heads of 17 subjects were exposed to 60 Hz, 200 μT MF delivered as 2 s pulses. Statistically significant evoked potentials were detected in EEG recordings during MF onset/offset transitions. The authors avoided pulse fronts through the use of a zero-switch.

A pseudorandomly slowly rotating (0.03–0.07 Hz) MF of the order of the geoMF in a triaxial exposure system of about 1 m in size altered pain perception and pain-related somatosensory evoked potentials in 11 subjects [107]. A two-meter cubic magnetic exposure system was used in [102] to expose subjects to an MF of the order of the geoMF. The α-rhythm of the EEG was measured in response to MF rotation in the horizontal plane. A response was observed when the static vertical MF was directed downward but not upward. The authors suggest that this rules out all forms of electrical induction, including electrode artifacts. The effects exhibited pronounced individual variability.

The ability of 34 men to perform magnetic orientation was tested in [108] using a 1.9 m system described in [101], consisting of three pairs of Helmholtz coils. The authors found that participants correctly indicated the direction of magnetic north in the absence of sensory cues—particularly with their eyes closed—if they had been previously exposed for several minutes to the geoMF without changing its orientation. However, if pre-exposure was conducted in a hypoMF, subsequent correct orientation was not achieved.

**Figure 4:**
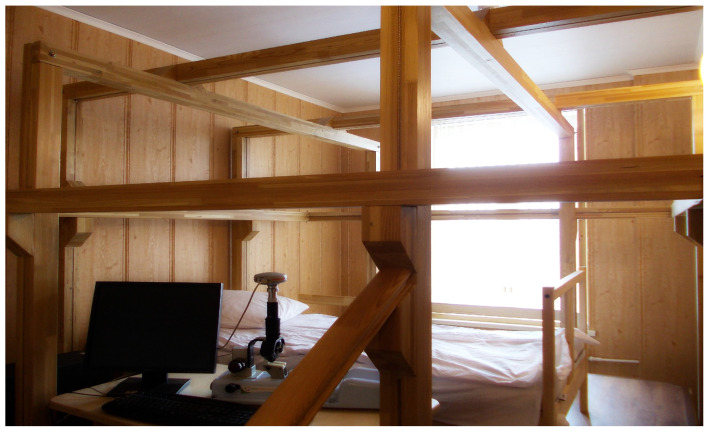
The *Faraday* magnetic exposure system, designed for monitoring local magnetic fields, particularly for recording and reproducing magnetic storms under laboratory conditions.

During the *Serena-Magnet* research project, a team from the Institute of Space Medicine (now the Federal Research and Clinical Center for Space Medicine and Biology, FMBA) in collaboration with the Scientific and Clinical Center of Russian Railways (OAO RZD) conducted pilot studies on the effects of combined simulated hypogravity and altered MFs on physiological changes in the human body [13]. The experiments were carried out using a 3 m cubic magnetic exposure system, *Faraday* (Figure 4), developed by one of the authors of the present paper [V.B.] and constructed under his supervision. For the first time, it was demonstrated that minor fluctuations in the geoMF are among the direct causes of the observed correlations between the geomagnetic activity and the physiological state of organisms [109].

A 28 μT, 60 Hz MF impaired sleep quality (measured via multiple EEG metrics) in 24 young males—see, e.g., [110,111] and other publications by this research group. The exposure system from [96] was used. In other limited studies with this same exposure system, the effects of geoMF alterations remained inconclusive [112] or were undetectable [99].

In [113], 20 participants were involved in a study on the effects of a 5 μT sinusoidal MF with a frequency simulating the α-rhythm. The experiment was conducted against the background of the geoMF; however, it was within a poorly described exposure system consisting of three rectangular coils measuring 2.9 m by 2.5 m. The results were interpreted as a statistically significant influence of the MF on the subjects’ emotional state, which was established in a blind experiment involving EEG recordings and participants’ self-reports of their sensations.

The above summarizes the effects of weak magnetic fields (MFs), distinct from hypoMFs, on the human body. Information on the effects of hypoMFs on the human body remains particularly scarce [114,115], despite the clear practical significance of this phenomenon. Generating a controlled hypoMF in a volume with a characteristic size exceeding that of the human body is technically more challenging than producing an MF at the geoMF level. Notably, the most pronounced biological effects of hypoMFs emerge at levels around 100 nT [31]. This necessitates suppressing natural and anthropogenic geoMF fluctuations, which often exceed 1 μT and can thus “spoil” the hypomagnetic conditions.

Exposure to hypoMF appears to affect human cognitive functions. In one of the first studies of this kind [93], an eight-meter system of rectangular coils was employed. Four subjects remained in its central zone for three weeks. No changes in physiological characteristics were observed. The response of one cognitive function to week-long exposure to hypoMF reached 30% in three subjects. However, in another publication by this author [116], changes in physiological and behavioral characteristics were not observed. The level of MF fluctuations, which often exceeds the residual constant MF in the exposure zone, was not measured. This might explain the absence of physiological responses.

In [117,118], a series of experiments placed subjects’ heads in a 1 m magnetic exposure system that generated either a geoMF analog of varying orientation or a hypoMF. The visual system’s sensitivity to light flashes on a screen was measured, with a statistically significant change of up to 7% depending on the orientation of MF relative to the screen. In a cycle of studies [119,120], the influence of hypoMF (<0.4 μT) on cognitive performance was investigated in 40 subjects. Exposure to hypoMF for 45 min induced statistically significant changes in five out of eight measured parameters, with effect sizes ranging from 1.3 to 6.2%. Under hypoMF conditions, error rates increased and task completion times lengthened. A near-statistically significant change in pupil area was also observed in hypoMF [121,122].

**Figure 5:**
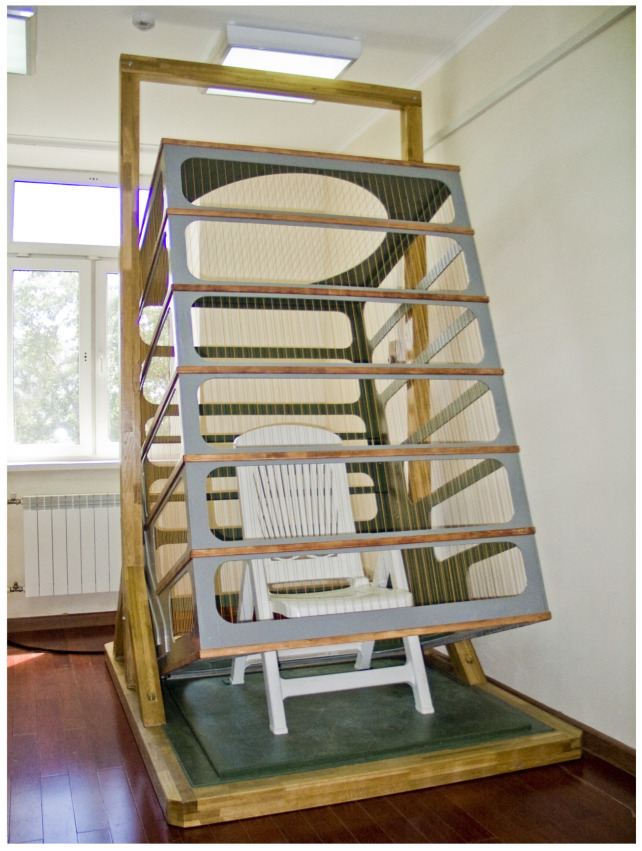
The *Arfa* magnetic exposure system designed for investigating the effects of weak MFs and hypoMFs on the human body under laboratory conditions.

In [123,124], it was demonstrated that exposure to hypoMF in the *Arfa* system (Figure 5) induced statistically significant alterations in capillary blood flow velocity, systolic and diastolic blood pressure, and heart rate variability features in elderly subjects.

At the same time, ref. [103] did not find similar effects. This study employed the same *Arfa* exposure system as previous works where the effects were observed. However, the system had been relocated to a different building. It should be noted that the *Arfa* system only compensates for the MF and its fluctuations along one axis. For the other axes, the MF magnitude is minimized solely by aligning the exposure system’s principal axis with the averaged geoMF vector. Fluctuations of the local MF along perpendicular axes remain uncompensated. The fluctuation level varies significantly depending on the system’s location and can reach microtesla-scale magnitudes in urban environments. The presence of residual MF fluctuations implies that the generated hypoMF intensity may vary by orders of magnitude depending on the installation site, which undoubtedly affects the reproducibility of the observed magnetic phenomena.

In [125], eight male subjects were examined under double-blind conditions after exposure to hypoMF, about 100 nT, in the *Arfa* system for up to 24 h (though see the above-mentioned caveat). Forty-eight biochemical parameters were analyzed from venous blood samples. The study demonstrated that exposure induced physical fatigue without pronounced subjective symptoms. Additionally, dried blood spots were chromatographically examined [126]; these data indicated either no hypoMF effect or statistically insignificant results due to insufficient sample size.

According to a recent publication [127], a 24 h exposure to the hypoMF in *Arfa* induced drowsiness in six test subjects compared to placebo conditions, with high statistical significance. A study [128] exposed six male subjects to the same hypoMF in the *Arfa* system for 32 h. The paper lacks explicit statistical hypothesis formulation and data processing details, undermining the reliability of conclusions. However, the reported differential effects of hypoMF on subjects with predominant sympathetic vs. parasympathetic tone appears plausible. This warrants verification using next-generation adaptive exposure systems with full three-axis compensation of the MF fluctuations.

The results described above are summarized in the table below, which also includes data on the effects of other types of MF exposure on the human body. These exposure types are hypoMF (hypo), extremely low-frequency sinusoidal (ELF), variations of the geoMF (var), and pulsed (puls) or static (stat) MFs. The table is structured as follows. Column Exp specifies the above exposure types; the exposed object—whole human body (B) or head (H); and the presence (y) or absence (n) of a magnetic effect, with these codes separated by spaces. Column Property indicates the investigated property and the measured quantity. Column MF provides the MF magnitude in μT, exposure time, and number of test subjects, separated by hyphens. Subsequent columns contain descriptions of the results, comments, and the publication reference.

The reported changes in measured parameters across the studies cannot be unequivocally linked to a specific “magnetic effect magnitude.” This is because authors often do not define this index in a general way, typically limiting their reporting to relative changes in the measured quantity. Given the diversity of both the measured parameters and exposure regimens, a unified definition applicable to all studies is only feasible in a formal sense. An attempt at such a general definition was undertaken in [31]. However, it proved unproductive: the effect magnitudes calculated from the data reported in the publications showed no correlation with any potential influencing factors such as the MF magnitude, exposure time, or others.

Consequently, we have limited ourselves to reporting the presence or absence of a magnetic effect. In concluding that an effect was present, the authors of all the cited works indicated a statistical significance of at least p<0.05.

Effects of ELF, static, and intermittent MFs are included for the sake of completeness. It is highly plausible that a single molecular mechanism is responsible for the emergence of all biological effects of weak MFs. In particular, according to the RPM, the effects of ELF MFs, as well as those of zero-switch pulsed MFs, are quantitatively almost indistinguishable from the effects of static MFs. This is because the RPM lacks frequency selectivity in the ELF range, and there are no known structures in the body capable of resonating at such frequencies.

A characteristic amplitude of 0.1 μT is given for geomagnetic variations. These observational data were included in Table 2 because, according to [129], minor variations of the geoMF with amplitudes from the hypomagnetic interval can induce effects analogous to those of hypoMF. This is plausible when considering the intrinsic rotations of large molecular fragments that carry the molecular targets of the MF.

It should be noted that, due to the existing uncertainty regarding the factors controlling the appearance of magnetic effects, the precise values of the measurable physicochemical quantities and spatiotemporal conditions are not yet of major significance. Furthermore, many studies contain different experimental series with varying numbers of participants, different MFs, and different exposure times. Accordingly, for each publication in the Table, the factors amenable to numerical representation are described using values characteristic of the given study, order-of-magnitude estimates, or semi-quantitative descriptions.

Approximately half of the publications on the study of human biological functions in ELF MFs report an absence of magnetic effects. This, however, does not mean that hypoMF should not elicit a magnetic effect. A substantial difference between exposures to moderate ELF MF and hypoMF lies not in the frequency but in the magnitude of the MF. According to [48,132], relatively large MFs on the order of the geoMF induce excessively fast precession and, consequently—unlike hypoMF—may not be accompanied by observable effects.

It is noteworthy that despite the existence of more than 10 large-scale systems (see Table 1) suitable for whole-body human magnetic exposure, only 14 experiments on the effects of hypoMF have been conducted since 1967. Of these, four reported negative results, and nearly all of these studies possess methodological limitations.

However, based on the data in Table 2, it can be argued that the effect of weak MFs on the human body is a well-established fact. At the same time, and consistent with the findings of [31], no specific correlations are evident among the numerical data presented. This suggests that the factors controlling the manifestation of the magnetic effect have yet to be identified. This contradictory situation appears to be linked to the unresolved fundamental problem in magnetobiology—namely, the lack of a recognized molecular mechanism underlying the observed effects.

## 5. Molecular Mechanisms of hypoMF Effects

A detailed analysis of potential molecular mechanisms underlying the effects of hypoMF is presented in [31]. The influence of MF on biological systems must, by necessity, involve interactions with the magnetic moments of atoms and molecules. This fundamental constraint helps identify the most plausible mechanisms among numerous proposed hypotheses. Currently, the most extensively studied mechanism is the radical pair mechanism (RPM) in spin chemistry [133,134], where MF modulates the spin state of electrons in transient radical pairs [135,136,137].

A key limitation of this mechanism is its low sensitivity to MFs, which is insufficient to explain the observed effects. Consequently, alternative models have been proposed, including those involving single abstract [48] and nuclear [138] magnetic moments, as well as the orbital motion of molecular groups within proteins [31]. Another hypothesis suggests MF action on the macroscopic magnetic moments of endogenous nanoparticles, which are present in certain organisms. However, this explanation is implausible for systems such as plants and some bacteria, where magnetic nanoparticles are absent despite observed MF responses. Notably, many earlier models—relying on the Lorentz force, cyclotron resonance, and parametric resonance—are now largely of historical interest.

The foundation of any molecular mechanism underlying the biological effects of MFs lies in the precession of microscopic magnetic moments [48]. This explains why both the absence of MF and exposure to strong MF produce biological effects of comparable magnitude. In a hypoMF, precession frequency is negligible, while in a higher MF, it becomes so rapid that chemical processes cannot track its motion. The system therefore responds to the time-averaged orientation of the magnetic moment, which is effectively static. Thus, in both scenarios, the microscopic outcome is identical—the absence of detectable precession—resulting in similar biological consequences. Certainly, in a *very* strong MF, other spin effects can emerge.

The widely discussed RPM involves the precession of two electrons in a radical pair relative to each other. A chemical reaction occurs only if the electron spins attain a specific *mutual* orientation. The quantum mechanical description of this process is more nuanced.

**Figure 6:**
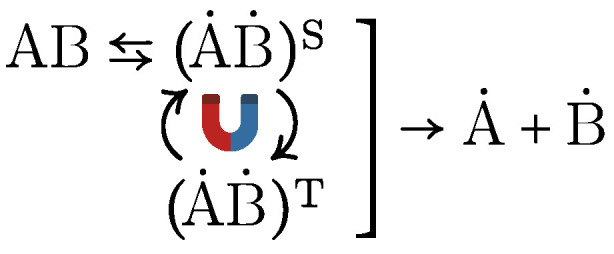
Breaking down of a neutral molecule AB into two radicals $\dot{A}$ and $\dot{B}$. Under the influence of an MF, the intermediate radical pair $\dot{A}\dot{B}$ undergoes intersystem crossing between the singlet ${\left(\;\dot{A}\dot{B}\right)}^{S}$ and triplet ${\left(\;\dot{A}\dot{B}\right)}^{T}$ states.

The RPM can be illustrated by the dissociation of a neutral molecule AB into two radicals, $\dot{A}$ and $\dot{B}$, forming an intermediate radical pair $\dot{A}\dot{B}$, as shown in Figure 6. Under the influence of a MF, this intermediate undergoes intersystem crossing between its spin states—the singlet ${\left(\;\dot{A}\dot{B}\right)}^{S}$ and triplet ${\left(\;\dot{A}\dot{B}\right)}^{T}$. Recombination into the parent molecule AB is spin-selective. Due to the conservation of angular momentum and the Pauli exclusion principle, recombination occurs exclusively from the singlet state. Consequently, the chemical fate of the radical pair depends critically on its spin state. If the correlated (coherent) spin state of the pair persists sufficiently long, the MF—by modulating the intersystem crossing rate—alters the yield of reaction products.

However, these intriguing effects emerge only when the precessional motion remains coherent—undisturbed by random thermal fluctuations. In quantum terms, this requires the radical pair system to be well-isolated from environmental decoherence. More precisely, these effects are critically dependent on the spin coherence relaxation time τ of the system.

A fundamental universal relation exists, γHτ∼1, that connects the critical (threshold) MF *H* with the electron’s properties and its environment—specifically, the gyromagnetic ratio γ and the thermal spin relaxation time τ [139]. When this field strength is reached, qualitative changes occur at the quantum level, which inevitably affect observable biological variables through biochemical cascades. The RPM takes into account both the electron pair spin dynamics and the chemical kinetics characterized by rate κ. Under reasonable idealizations, the generalized fundamental relation for RPM takes the form

(1) γHτ∼1+κτ

Here decoherence and chemical kinetics compete.

Magnetosensitive radical pairs occur in proteins such as cryptochromes [140,141,142] and photolyases [143]—ubiquitous protein molecules involved in biological clocks, DNA repair, growth processes, and other functions [144]. In these proteins, the kinetic rate $\kappa ∼1\;\mu s^{-1}$. The plausible range for the spin coherence time τ is 3–30 ns [137], making κτ≪1. According to Equation (1), τ therefore determines the minimum MF $H_{\min}=1/\gamma \tau$ required for observable RPM-mediated biological effects.

For τ values in the indicated range, $H_{\min}$ corresponds to 2–20 G (200–2000 μT). However, this threshold is insufficiently low for biological magnetic orientation, which requires sensitivity at the 1–2 μT level. Even for detecting variations comparable to the geoMF, the minimum detectable field must be nearly an order of magnitude smaller.

The situation is, in fact, more complex, as the aforementioned constraint represents only one of the necessary conditions for observing a magnetic biological effect. Another critical condition concerns the magnitude of the effect, which must exceed random fluctuations in the measured quantities.

Empirical evidence suggests that intrinsic stochastic and quasi-stochastic fluctuations—arising from biological uncertainty and superimposed biological rhythms—typically exhibit relative amplitudes ranging from 0.05 to 0.2 (5–20%) for measured biological parameters. For reliable detection, the primary RPM-mediated magnetic signal—manifested as a relative change in the yield of RPM reaction products—must surpass this noise threshold. The key question remains: is this condition satisfied?

The magnitude of the relative magnetic RPM effect for the specified range of τ values and at geoMF levels was determined in [139]. For the case where κτ≪1, the effect magnitude is approximated by

(2) $$M_{a}\left(H,\kappa ,\tau \right)=\frac{1}{5}{\left(\gamma H\tau \right)}^{2}\kappa \tau$$

This expression incorporates the ansatz γHτ from Equation (1) and comprises a product of two dimensionless factors: ${\left(\gamma H\tau \right)}^{2}$ and κτ/5. Both factors are substantially smaller than unity under typical magnetobiological conditions, since $H∼H_{\mathrm{geo}}$, $\kappa ∼1\;\mu s^{-1}$, and τ<50ns. Numerical estimates yield $M_{a}≲0.002$.

However, as noted above, the effect must reach at least 0.05 to be detectable. This orders-of-magnitude discrepancy becomes even more striking when considering biological responses to MF variations as minute as hundredths or thousandths of the geoMF, such as in animal magnetic navigation or organismal responses to geomagnetic storms.

Equation (2) can be solved for τ to provide a rough estimate of τ from experimental values of the relative effect in a given MF and known chemical kinetics rates. For instance, using measurement results from cryptochromes [140], photolyases [143], and human cytochrome P450 reductase [145], we obtain a plausible range for τ between 3 and 30 ns.

What are the RPM predictions regarding hypoMF effects? HypoMF effects represent the same class of phenomena as MF effects, but with the reference point shifted from zero MF to geoMF $H_{\mathrm{geo}}$. From Equation (2), reducing the field from geoMF to hypoMF *H* yields a relative RPM effect $\left(1-H^{2}/H_{\mathrm{geo}}^{2}\right)M_{a}\left(H_{\mathrm{geo}},\kappa ,\tau \right)$, where the coefficient $M_{a}\left(H_{\mathrm{geo}},\kappa ,\tau \right)$, as estimated above, is on the order of $10^{-3}$. This maintains the fundamental challenge in explaining the frequently observed hypoMF effects that are substantially larger in magnitude.

Other potential molecular mechanisms for biological hypoMF effects are examined in [31]. Their key properties are summarized in Table 3.

As shown in [139], the constraint on the dimensionless quantity γHτ arises from the fundamental energy–time uncertainty relation in quantum mechanics. Table 3 shows that this quantity determines the minimum MF required to observe magnetic effects across all mechanisms. Since the gyromagnetic ratio γ is known for potential primary targets, the minimum field is determined by thermal decoherence time τ, which remains unknown for MF targets under realistic conditions. Only order-of-magnitude estimates are currently possible.

For protein-bound radicals, the RPM effect (2) decreases rapidly as $\tau^{3}$ with diminishing τ [146]. Consequently, the magnitude of magnetic effects depends critically on τ. As said above, for plausible values of τ, the primary magnetic effect is extremely small, typically below $10^{-3}$ in magnitude. Thus, a fundamental challenge common to all RPM-based magnetobiological mechanisms emerges: the changes in magnetic moment dynamics induced by geoMF-level field variations are minute, while the observed biological effects are substantial, typically exceeding primary effects by 2–4 orders of magnitude. This implies the existence of amplification mechanisms inherent to living systems, distinct from inanimate chemical processes, e.g., [147,148,149].

A recently proposed amplification scenario [72] addresses the sensitivity limitation of spin–chemical mechanisms by enhancing weak primary chemical signals in response to external MF variations. The sensitivity can be increased by 2–3 orders of magnitude through the involvement of spin-correlated radical pairs in biopolymeric enzymes, particularly ribosomal enzymes. In this mechanism, the primary MF signal is transduced into elevated production of misfolded, nonfunctional, and often toxic protein aggregates. This imposes additional metabolic stress on protective systems and adversely impacts the speed and fidelity of cognitive processes. A key constraint of this scenario lies in its dependence on a highly nonlinear biochemical subsystem that triggers exclusively above a defined RPM signal threshold. As yet, no experimentally validated instance of such a subsystem has been characterized.

The explanatory potential of models such as the molecular gyroscope [132], or quantum rotor, remains incompletely understood. Quantum interference effect in rotating molecular groups exhibit a significantly higher sensitivity to MF variations compared to spin magnetic moments.

Indeed, it can be shown that a quantum rotor with a distributed electric charge—whose first *L* rotational states are populated according to a Boltzmann distribution—exhibits enhanced sensitivity to a MF, as described by relation

$$\gamma H\tau ∼\frac{1}{L}\;\left(1+\kappa \tau \right).$$

Comparing this with (1), one can observe that the minimum detectable MF for the quantum rotor is a factor of *L* smaller than in the RPM.

The mesoscopic quantum rotor serves as a model for, for instance, an amino acid residue being added to a synthesizing protein chain within the ribosome’s active site. After being released from the enzymatic “shackles,” the amino acid residue begins to rotate in a MF according to quantum laws. This coherent rotation persists until—as with electron spins in the RPM—it is destroyed by thermal decoherence and replaced by diffusive Brownian rotation.

For aspartic acid (Asp), L∼100. Preliminary estimates indicate that during the decoherence time τ, the residue rotates by several degrees in the geoMF. In a hypoMF, which is *L* times weaker than the geoMF, such rotation is absent. If this is linked to an increased probability of folding errors, it should produce an observable effect. Essentially, the mesoscopic quantum rotor acts as an interference amplifier for weak magnetic signals.

Although the RPM provides the only well-established molecular connection between MFs and chemical modifications, its inability to explain numerous biological effects of hypoMFs highlights the need for alternative mechanisms. The quantum rotor mechanism represents one such viable candidate awaiting further development.

## 6. Discussion

To date, no internationally recognized standards or regulations specifically govern exposure to hypoMFs in residential or occupational settings. This regulatory gap persists due to insufficient evidence in the scientific literature demonstrating adverse health effects from prolonged exposure to MFs significantly weaker than the geoMF.

However, the Russian Federation has implemented specific protective measures through its sanitary rules and norms (SanPiN 2.1.8/2.2.4.2489-09), which restrict continuous exposure to MFs four times weaker than the geoMF to no more than two hours daily. This regulation aims to mitigate potential adverse effects of hypoMF exposure in industrial, residential, and public spaces. Notably, the very existence of such restrictions implies official recognition of possible detrimental health effects from hypoMF exposure.

The available evidence presents a dual perspective. On one hand, only about ten independent research groups have investigated hypoMF effects on human organisms, yielding a modest number of publications. This limited dataset precludes robust classification or reliable generalizations, particularly given the absence of results replicated across different laboratories. On the other hand, extensive data exist regarding hypoMF effects on organisms in general. These data permit systematic classification by both physical [31] and biological variables [33,34], demonstrating that hypoMF effects manifest across virtually all organisms and organizational levels. This convergence of evidence suggests a fundamental biological phenomenon that demands thorough investigation, particularly concerning human organisms.

As evident from the preceding sections, data on hypoMF effects on human organisms remain inconsistent and lack sufficient reproducibility. Of the 14 studies investigating hypoMF exposure in humans, four reported no significant effects. When the results are pooled under the assumption of a single, homogeneous population, the combined *p*-value is roughly 0.3, which is above the significance threshold of 0.05. By standard statistical convention, the null hypothesis positing a random origin for “n”-type results must be rejected. However, it must be emphasized that the biological effects of MFs fundamentally violate the assumption of statistical uniformity underlying the standard statistical testing. They *typically* exhibit significant stochastic variability and individual specificity. As demonstrated in [88,106,150], MF effects on humans show pronounced individual variation. Consequently, experimental datasets of measured biological parameters become inherently heterogeneous—that is, they cannot be treated as belonging to a single statistical population. This heterogeneity poses substantial methodological challenges: conventional statistical approaches prove inadequate in magnetobiology research, as simple averaging effects across test subjects or studies tends to yield an insignificant mean value with a large standard deviation.

Mean values cannot serve as reliable indicators of magnetic biological effects, as individual responses may even exhibit opposite signs across different subjects, and their variability often substantially exceed the group-averaged effect. Robust conclusions require specialized metastatistical approaches [29,151]. Without such statistics, the only tentative conclusion possible is that hypoMF exposure tends to produce adverse outcomes, particularly manifesting as diminished cognitive performance on average.

Test subjects exhibit multidirectional individual effects of up to 10% in visual analyzer sensitivity tests, error rates, and task completion times [119]. This is directly relevant to astronauts’ prolonged stays on base stations during future deep space missions, where the MF will be 100 or more times weaker than on Earth. HypoMF may pose a risk factor for astronauts and their operational performance. At the same time, mitigating the “hypomagnetic” risk, unlike radiation risk, does not appear to be an overly complex engineering challenge. Devices that simulate the presence of the geoMF are simple and can be integrated directly into an astronaut’s spacesuit. Evidently, the high degree of field homogeneity characteristic of the local geoMF is not a requirement in this case.

In [130], correlation relationships between geomagnetic activity and heart rate parameters of astronauts in microgravity conditions aboard *Soyuz* spacecraft and the *Mir* orbital station were investigated. The authors concluded that magnetic storms altered vascular tone regulation and affected autonomic balance both during their occurrence and in the following 24 h. The transition from the geoMF to hypomagnetic conditions represents a disturbance in the magnetic environment orders of magnitude greater than even the most severe magnetic storm. There is substantial evidence to consider hypoMF as a factor in deep space missions, which must be accounted for during mission planning and ground-based preparations.

Emerging evidence indicates that the influence of the MF on cells significantly alters the effects of ionizing radiation [66]. In other words, radiation and MF exhibit synergistic interaction. It is plausible that hypoMF may exacerbate the adverse effects of ionizing radiation, as well as microgravity and hypogravity. To date, the effects of pairwise combined exposure to these three factors on the human organism remain unstudied. Thus, the development of an experimental model for the combined impact of a hypoMF, hypogravity, and ionizing radiation constitutes a pressing scientific challenge.

Elucidating the nature of hypoMF effects and assessing the associated risks is challenging, as it requires generating hypoMF conditions in terrestrial laboratories and testing a substantial number of animals and human subjects. The following tasks should be prioritized in this research direction: (i) Analysis of existing methods and technologies for creating hypoMF environments comparable to those expected during crewed interplanetary missions. (ii) Development and implementation of experimental setups for human and small laboratory animal exposure to hypoMF. (iii) Experimental investigations assessing cognitive functions and physiological adaptations in humans under combined hypoMF and hypogravity conditions simulating crewed lunar missions. (iv) Experimental studies to clarify the mechanisms of hypoMF effects on living organisms. (v) Theoretical exploration of molecular mechanisms underlying the combined effects of factors characteristic of interplanetary manned expeditions.

Noteworthy are studies on the effects of hypoMF on gravity-induced changes, even in non-human organisms. These data enable more substantiated predictions of hypoMF effects on humans in deep space. As is known, microgravity decreases bone mineral density and causes bone loss. Studies in [152,153] examined the combined effects of a 300 nT hypoMF and simulated microgravity on femoral bone mass loss in rats. The hypoMF exacerbated bone mineral density loss and worsened its biomechanical properties. There was more iron accumulation in serum, liver, spleen, and bone than under separate exposure to microgravity or hypoMF. Furthermore, hypoMF inhibited the recovery of microgravity-induced bone loss [69].

Unlike physiological parameters, which are regulated by multiple feedback mechanisms maintaining homeostasis, cognitive characteristics appear unconstrained by homeostatic limitations. Consequently, MF effects on cognitive performance may produce more pronounced manifestations. In such cases, individuals might perceive themselves as making autonomous decisions while remaining unaware that their decision-making capacity is being manipulated through specially configured MF. Preliminary experimental evidence supporting this perspective is available [154,155].

Thus, the study of the synergistic effects of hypoMFs and microgravity on human cognitive function appears to be of paramount importance. This necessitates a methodical progression: from the judicious application of existing data, to the establishment of specialized facilities with appropriate specifications, and finally, to the execution of experiments and their subsequent theoretical synthesis.

## 7. Conclusions

During extended lunar and Martian missions, astronauts will face chronic hypoMF exposure, the potential health consequences of which remain a critical area of uncertainty. Evidence from biological systems across multiple taxa—from microorganisms to mammals—confirms that hypoMF is a physiologically active agent. Crucially, rodent models demonstrate that hypoMF can exacerbate microgravity-induced osteoporosis and impede bone recovery, a finding with direct implications for spaceflight medicine. However, significant gaps persist in human research, particularly regarding chronic exposure. The current literature is insufficient to determine whether prolonged hypoMF exposure alters cognitive function or poses other significant health effects to astronauts.

Despite these knowledge gaps, the aggregate data suggest that prolonged hypoMF exposure is a plausible risk factor for crew health and performance, warranting its consideration alongside established challenges like hypogravity and radiation. In this context, the precautionary principle—as a rational risk management strategy—justifies further targeted research.

Therefore, investigating the effects of chronic hypoMF exposure in humans and developing potential countermeasures must be considered a critical priority for space medicine. This research is essential for mitigating potential health risks and ensuring crew performance on future interplanetary missions.

## Figures and Tables

**Table 1:** Characteristics of human whole-body MF exposure systems.

System	S ^1^	N	P	M	C	Reference
Naval Aerospace Medical Institute, USA	8	3	SL	H	−	[93]
Faculté de Médecine Pitié-Salpêtrière, France	1.2	3	L	AC	−	[94]
Swinburne University of Technology, Australia	2	2	S	G	−	[95]
Midwest Research Institute, USA	4	3	SL	G	−	[96]
Lawson Health Research Institute, Canada	1.5	3	S	AC	−	[97]
Institute for Occupational Physiology, Germany	1.8	1	L	AC	−	[98]
National Institute for Environ. Studies, Japan	2.7	3	SL	G	−	[99]
Institut de Recherche d’Hydro-Québec, Canada	1.6	1	S	AC	−	[100]
Kyungpook National University, Korea	1.9	3	S	G	−	[101]
California Institute of Technology, USA	2	3	S	G	−	[102]
Scientific Clinical Center RZD, RF, *Faradey*	2.7	3	SL	G	+	[13]
Institute of Biomedical Problems, RF, *Arfa*	1.2	1	S	H	+	[103]

^1^ S—size of the smallest coil frame, m; N—number of axes; P—position of the subject: S—sitting, L—lying; M—MF type: H—static hypomagnetic, G—geomagnetic-like, AC—weak alternating; C—type of MF compensation: − passive, + active.

**Table 2:** Experimental and observational data on human exposure to various magnetic fields.

Exp	Property	MF	Result	Comment	Ref
hypo B y	cognition/ vision	0.05-week-4	CVS measurands in four subjects in hypoMF have shown no effect. Critical flicker frequency of the visual system gave a 30% effect in three subjects at *p* < 0.001.	No compensation of the MF variations. Doubtful hypoMF value because of uncontrolled MF variations.	[93]
hypo B n	cognition/ vision	0.05-10 days-24	Neither the functions nor the behavior of man changed significantly during a two-week exposure to MFs below 50 nT.	There were no compensation of the geo and technogenic MF variations.	[116]
hypo H y	vision/ threshold	1-15 min-55	The photopic sensitivity of the human visual system decreased by 6–7% in hypoMF, $p<10^{-4}$.	The hypoMF magnitude was not quantified; however, based on the reported measurement accuracy, it should not exceed 1 μT.	[118]
hypo B y	cognition/ performance	0.4-44 min-40	HypoMF increased the number of errors and the time required to perform tasks in cognitive tests; an average effect 2.1% for all tests, *p* < 0.002.	Feedback compensation of the MF technogenic variations over z axis of the exposure system only. The standard MF deviations over x–y axes might be about 0.4 μT.	[119]
hypo B y	cognition/ performance	0.4-44 min-40	The effect magnitude in average was 1.49% at *p* < 0.004.	Elimination of the data falling out of the “three-sigma” range had no influence on the existence of the effect.	[120]
hypo B y	cardio/ HRV	0.4-1 h-32	HypoMF exposure demonstrates a clear effect on CVS and microcirculation, p<0.0003.	The MF effect manifests specifically 40–60 min after the onset of hypoMF exposure.	[123]
hypo B y	cardio/ hemodyna- mics	0.4-8 h-8	Resting in a hypoMF led to a significant reduction in heart rate and blood pressure in most cases.	No compensation of the x–y MF variations, no statistical treatment.	[124]
hypo B y	behavior/ orientation	1-few min-17	Groups of subjects were classified with different magnetic orientation tendencies. Magnetic orientation of the subjects was sensitive to the wavelength of incident light.	No information on hypoMF; it is assumed to be about 1 μT conditionally. Overcomplicated design of experiment difficult for interpretation.	[108]
hypo B n	blood/ proteome	0.1-24 h-8	Chromatography and mass spectrometry analysis of dry blood spots: results either mean the absence of the hypoMF effects, or they are unreliable due to small number of subjects.	No description of MF values and the way of MF measurements.	[126]
hypo B n	physiology/ EEG, HRV, etc.	0.4-8 h-8	Experiment, conducted at time periods with natural MF variations within 40 nT, did not reveal significant changes in EEG, cognitive tests, and auditory evoked potentials.	No compensation of the technogenic x–y MF variations, small sample.	[103]
hypo B y	blood/ biochemistry	0.1-24 h-8	A hypoMF significantly altered multiple venous blood biochemical parameters, p<0.05.	HypoMF z-component was reported to be about 0.1 μT. However, there were no compensation of the x–y MF variations.	[125]
hypo B y	cognition/ drowsiness	0.4-24 h-6	Based on self-assessment surveys, an increase in daytime sleepiness level was observed under the hypoMF in 72% of observations, p<0.003 by sign test.	The z-component of the hypoMF was 50–140 nT at no compensation of the x–y MF variations.	[127]
hypo B y	cardio/ ECG	0.4-32 h-6	In volunteers with predominance of sympathetic modulatory effects, the CVS response was significant in hypoMF lower than 0.15 μT.	No statistics, no hypoMF values, no compensation of the x-y MF components.	[128]
hypo B n/y	vision/ pupil size	0.4-44 min-40	The pupil size increased in the hypoMF. Effect of 1.6%, p<0.07.	Almost insignificant hypoMF effect. Inter-individual variability among participants was accounted for in the analysis.	[122]
ELF H y	behavior/ reaction time	1100-5 min-30	MFs can significantly affect reaction time performance.	Nonuniform MF 0.2 Hz.	[84]
ELF H n	behavior/ reaction time	1100-5 min-24	MF had no effect on reaction time at any time during the exposure.	Replication study. ELF 0.1–0.2 Hz superimposed on the geoMF. Nonuniform MF.	[85]
ELF B n	blood/ melatonin	10-9 h-32	The levels of serum melatonin and its metabolite in urine in exposed men did not differ significantly from those in sham-exposed subjects.	15 s pulses of the ELF MF by zero-crossing switch, linear polarization.	[94]
ELF B y	behavior/ pain	50-2 h-11	The reduction in the amplitude of pain-related stress-induced analgesia was observed after MF exposure.	Pain perception threshold, p<0.05, ELF 0.05 Hz. Incomplete description of the position of human in the exposure system; nonuniformity; incomplete data on the MF.	[107]
ELF H n	vision/ discriminati- on	100-8 min-99	MF exposure had an insignificant effect on the accuracy of estimating the duration of light flashes.	50 Hz intermittent 1 s on 1 s off, zero switch, nonuniform MF.	[86]
ELF B y	blood/ melatonin	20-2 h-30	MF exposure resulted in a half-hour delay in the onset of increased night-time melatonin concentration.	15 s pulses of the ELF MF by zero-crossing switch, circular polarization, p<0.05.	[95]
ELF B n	physiology/ melatonin, HR	200-8 h-7	Salivary melatonin levels determined hourly did not reveal alterations that can be related to the MF effect.	Sinusoidal MF 16.7 Hz. Large individual variability of the onset and amplitude of the hormone level and small sample could mask the MF effect.	[98]
ELF B n	blood/ hormone	20-12 h-10	No significant difference in the levels of four plasma hormones between blood samples collected during nights with MF exposure and those under control conditions.	Superimposed harmonics and 1-kHz 100 μT-amplitude MF transients per sec make interpretation difficult.	[99]
ELF H y	brain, cardio/ EEG	80-1.5 h-40	Alpha activity after 80 μT 50 Hz MF exposure doubled compared to sham.	Significance p<0.05, blood pressure and HR were not changed. Pain threshold was almost insignificant.	[87]
ELF H n	cognition/ performance	400-30 min-74	No significant difference was found in cognitive performance, psychological and physiological parameters.	50 Hz, continuous nonuniform MF. Large MF intensity might cause the absence of the effect.	[89]
ELF B y	physiology/ EEG	5-1 h-20	MF of 0–5 μT 8–12 Hz (DC-offset sinusoid) changes self-reported emotional state.	No details about exposure system, no information on the geoMF.	[113]
ELF H n	behavior/ vestibular balance	50,000-25 min-22	The parameters of human standing balance was analyzed to investigate postural modulation. There were no significant effects of the time-varying MF.	Nonuniform 20–160 Hz MF, 5 s pulses	[91]
var B y	cardio/ hemodynamics	0.1-24 h-27	MF storms affect regulation of blood circulation in cosmonauts during the flight dependently on the state of vegetative regulation.	Daily means of the most of HRV indices in control and exposed to MF storms groups differed at p<0.05. This seems to exclude indirect effects via radiation, electric fields, etc.	[130]
var B n/y	cardio/ rate of admissions	0.1-days-2000	Two days after a solar proton flux event that going till MF storm, the risk of admission for myocardial infarction increased by 54%, p<0.05.	What is the factor of influence, MF or protons? About 2000 persons. MF >80 nT converted from Ap index.	[10]
var B y	physiology/ hormone	0.1-days-900	Cortisol level grows with the geomagnetic activity at summer and autumn, p<0.05.	Observational study, geomagnetic activity is assumed to mean MF 0.01–0.1 μT.	[11]
var B y	cardio/ HRV	0.2-24 h-9	Artificial MF storm tends to randomize normal-normal RR intervals and decrease capillary blood velocity.	Incomplete statistics: no p-value for final statistical statement.	[109]
var B y	cardio/ HRV	0.2-22 h-8	Correlation of RR intervals with the Bx and By components of the MF was significantly higher during the artificial MF storm then in control, p<0.001.	Averaged RR interval and capillary blood velocity were not sensitive to MF storms. Small number of tested persons; RR to Bx correlation might be a selection bias.	[13]
var B y	cardio/ rate of admissions	0.1-days-4000	Risk of acute coronary syndrome in obese patients is associated with the MF storms, p<0.008.	Daily MF variations >80 nT (converted from Ap index), about 4000 persons.	[14]
var B y	physiology/ mortality	0.1-days-4,000,000	A one-standard-deviation increase in the daily Kp index was associated with a 0.3% rise in total mortality in South Korea.	Cardio-vascular deceases, stroke, and myocardial infarction mortality across 237 administrative districts between 2001 and 2019.	[131]
puls B y	brain/EEG	28-8 h-24	Intermittent, but not continuous or sham exposure, was associated with less total sleep time, reduced sleep efficiency, increased time in stage II sleep, and decreased rapid sleep at p<0.04.	Zero-crossing switch used to form 15 s pulses of the 60 Hz circularly polarized MF at night sleep.	[110]
puls B y	behavior/ pain	200-30 min-70	MF exposure does not affect basic human perception, but can increase pain thresholds.	No data on the pulse waveform.	[104]
puls B y	behavior/ tremor	1000-5 min-24	In postural tremor, the proportion of oscillations at frequencies between 2 and 4 Hz was higher during the real than during the sham exposure sequence, p<0.05.	Pulses of the 50 Hz ELF MF, no description of pulse’s fronts.	[100]
puls H y	brain/EEG	200-1680 s-17	Human subjects responded to onset and to offset of 2 G, 60 Hz, 2 s MF pulses by exhibiting evoked potentials, p<0.05 in 16 of 17 subjects.	Zero-crossing switch to exclude the fronts of MF pulses.	[88]
puls H y	neuro/fMRI	200-15 min-30	The ELF MF produced by the fMRI procedure could induce electric currents and cause an increase in pain sensitivity.	Highly nonuniform MF pulses superimposed on 1.5 T fMRI MF. Complicated waveform of 5 s pulses impedes interpretation. Incomplete description of timing, time 15 min is conditional.	[105]
stat B y	behavior/ orientation	50-min-64	Blindfolded humans were able to orient toward home when subjected to displacement-release experiments.	Observational data. 86 trials, incomplete statistics, different number of persons in series of the experiments.	[79]
stat B n	behavior/ orientation	50-min-100	Attempts have been unsuccessful to replicate an ability of blindfolded humans transported from home to indicate the direction of displacement.	Near 200 trials, up to 100 persons	[80]
stat B y	vision/acuity	19-few min-8	Experiments show a delayed (about 1 min) reaction in night-vision acuity after the reverse of the horizontal MF component, p<0.01.	Large MF effect. No information on the speed of the MF reverse.	[43]
stat B n/y	brain/EEG	45-2 h-50	MF alterations had no effect on EEG parameters. However, EEG could possibly change hundreds of ms after the start of the 4 s recording.	Exposition up to 2 h, MF ±45 μT, or ±90 μT. Overcomplicated design of MF exposures.	[112]
stat H y	vision/ threshold	48-15 min-30	Correspondence between viewing and MF direction results in a significant decrease of the visual discrimination threshold.	MF 70° rotation. 4% effect at p<0.02. No information on the speed of the MF turn.	[117]
stat B y	brain/EEG	35-1 h-36	Following geoMF stimulation, a drop in amplitude of EEG alpha-oscillations occurred.	Rotation of MF, drop in alpha activity, mainly p<0.01.	[102]

**Table 3:** Putative molecular mechanisms of nonspecific magnetic biological response.

Mechanism	Critical MF Relation	Primary MF Target	γ/T^−1^s^−1^	τ/s	Critical MF Estimate	Link to Chemistry
RPM	γHτ∼1+κτ	electron	$1.76\times 10^{11}$	$10^{-8}$	0.6 mT	known
Abstract precession	γHτ∼1	not applicable	–	–	1/γτ	not applicable
Quantum rotor	γHτ∼1 γHτ∼0.01	aminoacid residue	$2\times 10^{6}$	0.1	5 μT 50 nT	suggested
Proton mechanism	γHτ∼1	proton	$2.68\times 10^{8}$	0.1	40 nT	suggested

## Data Availability

Not applicable.

## Abbreviations

The following abbreviations are used in this manuscript:

CVS	cardiovascular system
DNA	deoxyribonucleic acid
EEG	electroencephalography
ECG	electrocardiography
ELF	extremely low frequency
fMRI	functional Magnetic Resonance Imaging
FMBA	Federal Medical-Biological Agency
geoMF	geomagnetic field
HR	heart rate
HRV	heart rate variability
hypoMF	hypomagnetic field
MF	magnetic field
MRI	magnetic resonance imaging
RF	radio frequency
RPM	radical pair mechanism
RZD	Russian Railways (Rossiyskie Zheleznye Dorogi)
